# The Institutional Worker

**Published:** 1913-01-11

**Authors:** 


THE
### INSTITUTIONAL WORKER
Being a Special Supplement to "The Hospital"

### BUREAU OF INFORMATION.

### Rules for Correspondents.

1. Every letter, on whatever subject, must be accompanied by the coupon to be cut from the back cover (inside page) of The Hospital, current issue, and must contain the name and address of the correspondent with pseudonym for publication if desired.

2. Replies by post cannot be given, save under exceptional circumstances at the Editor's discretion.

3. Letters from Approved Homes in reply to special needs published in the Bureau should state terms and full particulars, and be sent prepaid under cover to the Editor of the Bureau with name written across coupon for identification.

4. Proprietors of Homes which have not yet been entered on the List of Approved Homes, but have spare accommodation likely to suit special needs, are invited to write for an application form for registration. The fee for registration, which includes two announcements of the Home in the Bureau, is 5s.

5. Special needs of every description relating to those who are sick or in difficulties are made known in these columns free of charge.

6. All communications to be addressed to The Editor of The Hospital, 28 Southampton Street, Strand, London, W.C., and marked "Bureau of Information."

### Approved Homes.

Note.—*Homes recently added to the Approved List after direct personal testimony from medical men and others to their good management.*

**London.**—Ferndale, Cranfield Road, Brockley, S.E. Principals: Mr. and Mrs. Louis Lardent. Comfortable Home for patients needing massage, electrical and rest treatment. Moderate inclusive terms.

### Homes Wanted.

**For Elderly Lady.**—The patient is a lady, aged eighty-nine, who broke her leg three months ago and is not likely to use it again. Means are limited. Easy access from Romford desired. Write stating lowest terms to M. R. (143a).

### Training and Employment.

**Dispensing in Egypt.**—If you have only a dispensing certificate and no general training, we fear you could not get employment either in a general hospital in Egypt or in a mission station. You could write to the Matron, Kasr-el-Ainy Hospital, Cairo, or to the Anglo-American Hospital, Gezireh, Cairo. In regard to mission work, consult the Secretary of the Church Missionary Society, 16 Salisbury Square, London. You do not say whether you wish for a paid post.—Dispenser.

**Clientele for Nursing Home.**—Absolutely impossible to offer any percentage to medical men. Such a proposal might lead to a practical boycott. You can write to all whom you think likely to recommend patients for such accommodation as you offer, and send a well got-up leaflet, giving photographs of the exterior and of one or two rooms. Intimate that, owing to your new start, you can receive patients at very reduced terms. Let all the general practitioners in that part of the county have the prospectus, and leave a copy with chemists and agents likely to be asked for Homes. Make friends in your locality. Keep your rooms in spick-and-span order, and show them to any likely to be interested. A standing advertisement in the local and other papers often helps.—Sister E.

**Worker with Slight Disfigurement.**—Can any of our readers help the following case, which commands much sympathy? The applicant, aged thirty-eight, is a skilful needlewoman, thoroughly domesticated, with a knowledge of dressmaking. She has suffered from facial tuberculosis, and, though she made a good recovery six years ago, she has sustained a scar which makes it difficult to obtain employment. She has been assistant in hospitals, and would like to get a situation as useful help at low wages in some institution or Nursing Home out of London. Replies forwarded to Edwardes.

### Needs and Helpers.

**Blind and Deaf.**—We acknowledge with cordial thanks the receipt of a donation of £1 from the Rev. J. Pullein-Thompson, Hon. Secretary of the National Blind Society, for this former hospital worker, now reduced to great penury through the loss of sight and hearing after contracting typhoid fever in the institution to which she was attached. We are glad to state that two subscribers have been secured through the Bureau of a guinea a year each for the purpose of nominating her for a pension from the above Society. It is hoped to make up a monthly 5s. for her until the pension becomes due, and any help, however small, in that direction will be gladly welcomed. Thanks are due to the reader who contributed a Christmas parcel. We hear from the patient: "This is a most Happy New Year for me; my nerves are better already, as I am not worrying over the future."

### Nursing in the Empire and Abroad.

**Island of Mauritius.**—The Colonial Nursing Association have a nursing staff in the island for private patients, and it is to them you should apply. There is a Civil Hospital at Port Louis, the capital, and here probationers receive a two-years' training, and appear to do well afterwards. The summer months, during which the heat is very oppressive and hurricanes frequent, are December to April. Malaria is now very prevalent, and on this account the island is considered less healthy than formerly. Boats go every four weeks from Southampton and every fortnight from Marseilles. French is more spoken than English. Very glad to afford more information if required.—L. A.

**Nurses for Blantyre.**—We would strongly urge you to await further information. Only a few days ago we received a communication from the principal medical officer at Blantyre, Nyassaland, stating that no private nurses were needed in this locality, as there was no scope whatever for them. After this definite statement we feel only the actual promise of a salaried post could render it prudent to attempt a practice in this locality.—Prudence.

**Prospects in Persia.**—The demand for trained nurses exists, but is limited as far as the European colony is concerned. Address your query to the Secretary of the Teheran Nursing Association, c/o H.B.M. Legation. This Association, membership of which is limited to the British and American residents, employs one trained nurse engaged through the Colonial Nursing Association, and unless you are encouraged to come by this authority it would be wiser to let it alone.—Ella P.

### Letter-Box.

**Communications have been received and attended to from:**—Mrs. M., Dublin; Nurse R. L., West Hill; Medical Officer, Blantyre, Nyassaland; Town Clerk, Liverpool; Miss L. W., Vyrnwy; Nurse E. R. M., Ireland; Miss E. A. K., Falmouth; E. J. M., Hackney; Mrs. K. M. A., Catford; Mrs. J. J. T., Hitchin; Mr. B. P.; Miss E. M. W., Streatham.

**Letters have been forwarded from:**—Miss G. Mrs. P.; Sister A.; Mrs. T.; Miss R. (2).

**The Editor is much indebted for testimonials received in response to inquiries from:**—Dr. J. S. Wilson; W. G. Dodd, Esq.; Miss Flood, superintendent; Miss Shrewsbury.

**The Editor begs to acknowledge the receipt of communications, with enclosures, from:**—Miss E., Llangollen; the Rev. J. Pullein-Thompson; Institutional; Miss A. W.

Many thanks for information *re* Turner Memorial Home, Liverpool:—Senior Superintendent, Royal Infirmary, Liverpool; also Miss Bateman.

Your first letter has just come to hand, bearing date December 24—evidently a victim of the Christmas post.—MRS. A., Catford.

We wrote to make an appointment for you to see us. Did you receive our letter?—EDWARDES.

Replies to Nurse S., Miss C. T. L., M. A. W., Miss E. I., Oswestry, and Gwalia will be given next week.

The second form was sent in error.—MISS E.

*** A Revised Edition of The Bureau Leaflet is now in the press, and will be ready shortly.

### Institution Facts and Figures.

### Selected Answers to December Questions.

THE MOST POPULAR SUPPER DISH.

1. EGG AND TOMATO PIE (without Meat).—Probable cost with eggs at contract price, 1s. a dozen, inclusive of sauce and condiments, 5s. 6d. for fifty persons. Quantities: 1 egg to each person, 4 tomatoes, $1\frac{1}{2}$ pint of milk, 2 oz. butter.

*Method.*—Blanch the tomatoes, skin and cut up into slices; boil the eggs hard, shell them and cut into slices; place a layer of tomatoes in the bottom of the pie-dish, then a layer of egg, and pepper and salt, and so on till the dish is nearly full—pour on the sauce and chopped parsley; bake for half to three-quarters of an hour.—BETTINA.

FISH MOULD.—Ingredients for fifty persons: 10 eggs, 9 lb. cod, $1\frac{1}{2}$ pint white sauce. Probable cost: Cod at 4d. lb., 3s.; eggs at 1s. dozen, 10d.; white sauce, 6d.; total, 4s. 4d.

*Method.*—Boil the cod, remove bones and pass the flesh through a sieve, add 1 lb. white breadcrumbs, a little chopped parsley, pepper and salt to taste, $1\frac{1}{2}$ pint of white sauce, the yolks of 10 eggs. Mix thoroughly, and either fill small moulds or one large one; bake until nicely set; turn out and serve with any fish sauce.—A. W.

CORNISH CUTLETS.—Any cold meat can be used, and one slice should be allowed to each person; potatoes 6 lb.

*Method.*—Take some thickish slices of cold beef or mutton (cold boiled mutton is good), trim them into heart or cutlet shapes; place them in a pie-dish; pour over them a little oil and vinegar, chopped parsley, pepper and salt, and let them steep in this mixture for one hour. Have ready some seasoned mashed potato, which must be cold before using, place on a smooth board—the reverse side of a pastry board will do—wrap each piece of meat in potato, taking care to retain the shape; dip in egg and breadcrumbs and fry in hot fat. The cost of this will vary from $1\frac{1}{2}$d. to 3d. per head, according to price of meat. It is a most excellent way of using up cold meat without cooking away its natural juice. If there are cold potatoes left they could be mashed with others to make up the required quantity.—CORNISH WOMAN.

BIRDS' NESTS.—Quantity for fifty persons: 1 egg to each person; contract price, 1s. a dozen, 4s. 3d.: 4 lb. sausage meat at 10d., 3s. 4d.; total, 7s. 7d., or rather less than 2d. a head.

*Method.*—Boil hard as many eggs as required; shell them and put by to cool. Take 4 lb. sausage meat, skin round, and allow to each egg the amount of half a sausage. Wrap the sausage meat round each egg so that it is completely covered, roll in flour, egg, and chopped, uncooked vermicelli, and fry a nice brown; serve cut in half and placed on the dish with the egg side uppermost.—POPULAR.

QUESTIONS FOR JANUARY.

1. STATE the method in use in your institution for making either beef-tea or broth, or both. How much does the liquor work out at per pint respectively? State the price paid for the meat, and what saving, if any, is effected by the use of a stock-pot.

2. What difference could you make in the bill of fare for the staff, irrespective of their numbers, if you were allowed an extra shilling per head per week for their board? The original allowance may be estimated at six, seven, or eight shillings per head. It is desired to demonstrate how much could be obtained in luxuries for the extra shilling.

The figures must be the result of actual observation and computation, not estimates. Either or both of the questions may be answered by each contributor. The best answers will be published, and for each contribution considered by the Editor to be of sufficient interest for publication payment of FIVE SHILLINGS will be made.

The following rules must be observed by contributors to the FACTS AND FIGURES Column:—

1. Answers must be written on one side of the paper. They must bear the name and address of the sender and be accompanied by coupon to be cut from the back cover (inside page) of the current issue of THE HOSPITAL. A pseudonym must be chosen if the name is not to be published.

2. Answers must be addressed to the Editor of THE HOSPITAL, 28-29 Southampton Street, Strand, London, W.C.; they must be marked in the left-hand corner "Facts and Figures," and must be received before the end of the month.

### Editor's Notices.

**Contributions:**

Contributions should be written, or preferably typed, on one side of the paper only, and all articles sent in are accepted upon the distinct understanding that they are forwarded to THE HOSPITAL only.

The Editor cannot undertake to return MSS. not used, but when a stamped directed envelope is enclosed the MSS. may be returned if a special request is made.

Accepted articles and paragraphs of news will be paid for after publication at the scale rate.

**Address:**

To prevent delay all contributions and letters on editorial business must be addressed exclusively to the Editor, "The Hospital," 29 Southampton Street, Strand, London, W.C. It is important that this regulation shall be strictly observed.

**Correspondence:**

Correspondence on all subjects is invited. The name and address of correspondents must be given as a guarantee of good faith, but not necessarily for publication.

**Special Articles:**

Special articles are invited, and questions, inquiries, and paragraphs upon all matters relating to the work, administration, and management of general, special, mental, fever, cottage and convalescent hospitals, sanatoria, homes, institutions, societies and organisations for the treatment or care of the sick, injured and dependants of all classes. Every matter affecting the interests and welfare of the staff of all grades working in these institutions will receive special consideration.

**Administrative Medicine, Research and Items of News:**

Special terms will be paid for approved articles on subjects in this wide field by experts who have made some section of it their special study and interest. We wish to give prominence to every movement tending to advance accuracy of diagnosis, efficiency in treatment, and the development of modern methods to eradicate disease and promote the welfare of the suffering.

**A Bureau of Information:**

We invite inquiries and applications for information and help in providing for that numerous class of sufferers who require special care or house-room which they cannot obtain in their own homes or secure for themselves. **We cannot, however, prescribe, or recommend practitioners.**

**Books for Review:**

Publishers are particularly requested to send advance proofs of any new books of importance whenever possible, as the Editor has made arrangements to publish immediate reviews on a new plan.

**Photographs, Plans, Blocks, and Illustrations:**

It is requested that wherever possible MSS. may be accompanied by illustrations in any of the above forms. The name of the sender and of the article to which it belongs should be written on the back of each photograph, drawing, or block for purposes of identification.

**Local Papers:**

Newspapers containing reports or news paragraphs should be marked and addressed to the Sub-Editor.

**The Coupon System:**

A coupon will be found at the bottom of the third inside page of the cover each week. This coupon must be attached to every question or inquiry to which an answer is desired in THE HOSPITAL.

### A Great Help.

IN consequence of the ever-increasing circulation of THE HOSPITAL it is often a matter of considerable difficulty to obtain a copy of the current issue at the local newsagents. In these circumstances we strongly recommend you to guard against the contingency of THE HOSPITAL being sold out by sending a subscription to the Journal either through a bookseller or direct to the Publishing Offices. By this means it is possible to secure a copy of the Journal almost immediately upon publication.

This is *a great help* when seeking a post, as it enables you to send in your application without delay, an advantage which may secure you the appointment. In addition to this, as a subscriber, no matter how many times you may change your address, or in what part of the country you may be at the moment, a postcard will ensure that you receive THE HOSPITAL on the same day and at the same hour as when at home.

**Subscriptions** may begin at any time, and are payable in advance. Cheques and Post-Office Orders should be crossed London County and Westminster Bank, Covent Garden Branch, and made payable to the Manager of THE HOSPITAL.

RATES OF SUBSCRIPTION (payable in advance).

UNITED KINGDOM.

| | | |
|---|---|---|
| Three Months (including Postage) | ... ... ... ... | 2s. 0d. |
| Six Months do. | ... ... ... ... | 4s. 0d. |
| Twelve Months do. | ... ... ... ... | 6s. 6d. |

FOREIGN AND COLONIAL.

| | | |
|---|---|---|
| Three Months (including Postage) | ... ... ... ... | 2s. 6d. |
| Six Months do. | ... ... ... ... | 5s. 0d. |
| Twelve Months do. | ... ... ... ... | 8s. 8d. |

### *SUBSCRIPTION FORM.*

*Please send me* THE HOSPITAL *for..............months, commencing with your issue of.............................., for which please find enclosed............................*

*Name*..........................................................

*Address* ..........................................................

..........................................................

*Date*..................................

*To* .................................... *Newsagent.*

### Manager's Notices.

**Letters relating to the publication, sale, and advertising departments of "The Hospital" must be addressed to The Manager, "The Hospital," The Hospital Building, 28 and 29 Southampton Street, London, W.C.**

**Advertisements:**

To ensure insertion, all advertisements for THE HOSPITAL must reach the Manager not later than Wednesday morning in each week. For scale of charges see pages v and 4.

**Remittances:**

**It is especially requested that remittances be made by Cheque or Postal Order, and in halfpenny stamps only when the amount is under sixpence.**

